# On the Classification of Mental Diseases
*Essai de Classification des Maladies Mentales. Par M. Baillarger, Médecin de la Salpêtrière. Paris. 1854.


**Published:** 1854-10-01

**Authors:** 


### Art. V.—ON THE CLASSIFICATION OF MENTAL DISEASES.*

The name of Baillarger is widely and favourably known to the psychological world. Trained in the school of Esquirol, he inherits in a remarkable degree the enlightened spirit of practical observation which distinguished that illustrious physician. He has long been, in conjunction with his able confrère, Brierre de Boismont, one of the editors of our continental contemporary, the *Annales Medico-Psychologiques.* His name is familiar to the British medical world through a course of Lectures "On Mental Alienation," delivered by him at the Salpêtrière, and edited by Dr. Barnes, one of his pupils, in the *Lancet* in 1844-5. M. Baillarger takes a foremost position by his zeal, his experience, his abilities, and his original researches, in the rank of those physicians who have of late years contributed to the improvement of the condition of the insane, and who most worthily uphold the honour of the profession in the great department of psychology. When, therefore, a physician occupying so honourable a position, and so well qualified for the task of instruction, announces an essay upon the classification of mental diseases,—that is, upon the very fundamental questions of psychological science,—our expectations are raised to a proportionate height.

Classification is at once the foundation and the highest expression of our knowledge. Classification, which in dogmatic teaching precedes and serves for an introduction to the study of a given science, must, in logical order, be the last enunciation, the final generalization of our previous knowledge of that science. Considered not in a dogmatic point of view, but in that natural and more instructive spirit which is content, with patient inquiry, to follow the gradual progress of knowledge resting upon slow accumulations of experience and the well-weighed deductions of reasoning, classification,—that is, a classification that shall be rigorously exact,—must be postponed until the last term of scientific investigation, until we have adequately mastered all the antecedent elements which are to be the subject-matter of classification. If this truth apply to science in general, it of course applies to psychology. We think it important to refer to this fundamental principle, because there exists a disposition, arising out of the natural indolence of the human mind and its consequent readiness to accept the dictum of authority as a substitute for knowledge, to look upon classifications not in the only true light we have indicated, but as *aide-*

* Essai de Classification des Maladies Mentales. Par M. Baillarger, Médecin de la Salpêtrière. Paris. 1854.

*mémoires*, as a sort of pocket compendium, containing in themselves the essence and concentration of all useful knowledge. That this error is especially prevalent in psychology, as well as in other departments of medicine, we have often occasion to deplore. Given a definition of insanity, given a definition of a delusion: and straightway many a man—not so often of the medical profession, as members of the bar or the general public—thinks himself competent to pronounce an unerring judgment upon the sanity or insanity of any particular individual. The tendency of slothfulness to accept definitions and classifications on authority, and the impulse of temerity to apply them in practice, if indulged, cannot but prove a serious discouragement to independent inquiry, a great obstacle to the progress of learning, and the source of many grievous practical errors. Admit that a given classification of mental diseases is perfect: for the minds of the majority this would be equivalent to an admission that the science of mental pathology is perfect; that the disorders of the mind have been explored and fathomed to their nethermost depths. Howsoever attractive a definition or a classification may appear, we need not say that we are as yet very far indeed from such a consummation.

But it may be urged that we cannot do without definitions and classifications. We will grant that *it is difficult* to do without them; that rightly used, not strained beyond their proper use, they are valuable aids and *guides* to the acquisition of knowledge. But they must be lightly worn. We must ever jealously keep watch, lest they usurp an undue dominion over our judgment. We must be ready at any moment to discard them, when we find they will not embrace all the facts they were made to include. They must be not our masters, but our most obsequious servants.

And yet the ambition to frame definitions, to construct classifications, has always influenced the great teachers of psychology. It springs in a great measure from the natural longing, that seizes upon the most patient inquirer, to arrive at the term of his labours, to reach that point where he may rest, and look behind him and contemplate what he has achieved. Many thus hasten on to the goal. It is given to few to reach it honestly. The course marked out by the hand of science is often broken and difficult. The asperities are not fairly overcome, but evaded: a short cut is not seldom preferred.

But to enter upon the task immediately before us. How far has the learned physician of the Salpêtrière succeeded in the solution of the problem? We may at once relieve ourselves from any attempt to inquire how far the essay before us, or rather the classification propounded in it, extends our knowledge of mental disease. The essay makes no pretension of this kind. The author is far too much versed in the daily practical difficulties met with in our intercourse with the insane to fall into the vulgar error we have endeavoured to combat, of supposing that the most symmetrical definitions can stand in lieu of exercised skill and an intimate acquaintance with minute details. The essay in question is an introductory discourse to a series of dogmatic and clinical lectures delivered at the Salpêtrière. He tells his pupils that he has "striven to render his classification as practical as possible, and to supply them with a method which should serve as a guide in their study of patients." This is the true spirit. On no other condition could we consent to listen to a classification at the *outset* of an extended inquiry into the nature and treatment of mental diseases. He does not, by his initiative classification, profess to prejudge the disputed questions in mental pathology. It is only advanced as an instrument, an *organon*, to facilitate inquiry. We may therefore proceed at once to consider the terms of his classification, and to discuss its merits and defects.

M. Baillarger begins, as every one begins who has a new theory to enunciate, by showing the fallacy of former theories. He assails all previous classifications with the general objection that they are based upon purely psychological data, and that they cannot be applied to clinical observation. He exposes the contradictions existing between the definitions proposed by M. Delasiauve and M. Guislain, in order to throw discredit upon what he calls the purely psychological method. Thus M. Delasiauve divides mental alienations into *intellectual* or general, and *sentimental* or partial. "But," says M. Baillarger, "in the first rank amongst intellectual alienations you place mania. Now, M. Guislain tells us that 'mania is a disease of the moral, apyretic, irresistible, in which there is exaggeration of one or more of the phrenic functions, characterized for the most part by a state of agitation, or sometimes by a manifestation of active or violent passions.' Thus, then, the kind of insanity which you regard as the type of intellectual alienations is precisely that which M. Guislain regards as a moral alienation." In this manner, certainly striking, but not free from error, M. Baillarger, by contrasting the definitions of two eminent physicians, and exhibiting their inconsistency with each other, thinks to overthrow all definitions based upon psychological distinctions.

Before stating his own classification, the author thinks it necessary to define the meaning to be attached to the words, *Folly (Folie)*, *Delirium*, and *Mental Alienation*. He has recourse at once to an example; that is, he substitutes an illustration for a definition. He says, "It is not rare, when an insane patient has recovered, to see him preserve a residuum of his disease. Thus we have at this moment a curious instance of this kind. A woman who was completely insane during seven or eight months, recovered several years ago, and now fulfils in the establishment, with considerable skill, a very difficult office. Nevertheless, she preserves a very serious symptom of her original affection. She remains subject to hallucinations of the sense of hearing; but she perfectly accounts to herself for the phenomenon she experiences. This is why I say that, being no longer mad or insane, she is nevertheless affected with a serious lesion of the intelligence." "When she was mad or insane, she had not the consciousness that her understanding was impaired, she did not appreciate the errors of her condition, or else she held them for realities; in a word, this woman was cheated by her malady. Now, on the contrary, she judges of her hallucinations in the same manner as the physician himself judges of them; she recognises them as sensations without object; the sick woman knows herself to be sick, and that is enough to make her no longer mad." . . . . "Let us rest, therefore, upon this point, that the lesion of the intelligence and the loss of the consciousness of this lesion, are two very distinct facts, and that both are necessary to constitute a true mental alienation."

M. Baillarger next anticipates an objection that might be urged. He admits that "there are patients possessing a perfect knowledge of their condition, and who are not the less insane. This is the case with persons subject to uncontrollable impulses ('*impulsions insolites.*')" In this case the distinction would be derived from the defective power of the will. "But," he continues, "uncontrollable impulses are not sufficient to constitute insanity." He cites the case of a man who, during twenty years, struggled against the impulse to kill a mother whom he tenderly loved. This man left his country in order to protect himself from the danger which threatened him. It was only after twenty years that this impulse to murder overcame the efforts of his will. The patient feeling himself conquered, applied to be restrained. From that moment there was insanity. Hitherto, the voluntary faculties were deeply affected, but the patient was not insane. "Thus the lesions of the intelligence and of the will are so distinct from insanity or alienation, that these lesions may exist without insanity or alienation. These distinctions teach us, moreover, that insanity has two sources; the first, which consists in the loss of the consciousness of the lesions of the understanding; the other, in the want of power to control certain impulses. . . . . It results from these distinctions that *insanity is the privation of free will, in consequence of a disorder of the understanding*. It is important to remark that free will represents both the integrity of the consciousness and of the will. Hitherto, two very different elements of insanity have not been sufficiently distinguished: the lesion, on one side, and the loss of consciousness on the other."

Is this definition of M. Baillarger's secure from the assaults of criticism? In the first place, is it free from that general objection with which he has himself attempted to demolish all previous definitions? Is it not psychological, like the rest? In the second place, is it new? Is it something essentially distinct from other definitions, or is it merely a variation in the mode of expression, in the manner of stating? In the third place, we will ask, What shall we gain by adopting it?

1. What is the fundamental character of this definition? It is drawn from the observation of the condition of the intellectual and moral faculties. It rests upon an appreciation of the integrity or impairment of the intelligence, of consciousness or judgment, and of the will. Is not such a method strictly psychological? Does it acquire a different character because the author lays it down as necessary to his definition that there should be the coincidence of *two* distinct mental phenomena,—the lesion of the intelligence and the loss of the consciousness of this lesion? The two phenomena are both psychological; shall the resultant third condition be other than psychological?

2. Is the definition new? We think not. Is there any notion of insanity more general than that which rests upon the distinction, so very clearly put by the author, between the lesion of the intelligence and the loss of consciousness or judgment? So long as a man is conscious that the sensations he is subject to are unreal, so long as he knows that his conceptions are false, so long is he held to be sane. When, on the other hand, he gives way to the belief that his morbid sensations are real, that he takes his false conceptions for true, that he does not perceive their absurdity or incongruity, then he is on all hands pronounced insane. That is what M. Baillarger says, and what has long been said by others. Nor are the terms or the form employed by M. Baillarger very different from those in common use. The definition of M. Baillarger is, therefore, psychological, and not new.

3. Our third question, What shall we gain by adopting M. Baillarger's definition? is pre-determined by our answers to the two preceding questions. It will continue to be useful as a guide to practical investigation. It must not be accepted as the ultimate expression of medical science. It must not be allowed to stand in the way of further inquiry. When M. Baillarger says that hitherto we have not sufficiently distinguished two very different elements in insanity—the lesion on the one hand, and the loss of consciousness on the other—we are ready to admit the general value of this analysis or resolution of the component parts of insanity. But some reservation is necessary. It is not always easy in practice to effect this analysis,—that is, not by the chronological process. Marked antecedence in time of the lesion, which M. Baillarger ranks first in order of importance and of development, over the loss of consciousness, cannot always be observed. Frequently they set in, or at least are observed, simultaneously; and frequently they disappear simultaneously. In logical sequence it may appear that at first sight M. Baillarger's theory, which assigns the first place in time and importance to the lesion, and the second to the loss of consciousness, is correct. But we may ask, is this the constant order of Nature? If we accept M. Baillarger's definition in the term he has employed—namely, *loss of consciousness* of the lesion, we give up the matter; we place it beyond dispute. The term we have quoted is a *petitio principii.* It is obvious that the lesion must pre-exist, or it could not be the subject of loss of consciousness. But if instead of accepting the term, loss of consciousness, we inquire whether that intellectual faculty which compares, judges, and decides, may not be diseased independently and primarily, what must our answer be? Are there not cases of insanity in which the disorder of the intelligence, by which M. Baillarger means the faculty of conception, plays but a secondary part, in which the lesion of the understanding, or of that faculty which, according to M. Baillarger, leads to the loss of consciousness, is primitive? Can we, with the experience of clinical practice present to our minds, yield our assent to this proposition, enunciated by M. Baillarger: "The lesion of the intelligence is the more important element for the physician; when you have cured this, you will well nigh have cured your patient"?

Taking a departure (which he thinks a secure one) from this definition, our author proceeds to develope his classification.. He complains that writers have, for the most part, dwelt far too much upon the general history of insanity, to the neglect of the more practical history of the forms of insanity. He observes very justly, "That we must carefully distinguish the alienation conceived in an abstract and general manner, as it is allowed to philosophers and magistrates to define it, from the alienation which comes under the observation of the physician, and which he has to treat." M. Baillarger, however, recognises the importance of studying the subject both generally and specially. The general pathology of insanity (he says) will comprise the physiology of delirium, and of all the other generalities which in methodical teaching precede the study of particular diseases. Under special pathology are ranged the description and the study of the different forms of mental alienation. General and special pathology, therefore, constitute the two leading divisions of M. Baillarger's methodical classification.

### MENTAL DISEASES.

### General Pathology.

*Elementary Lesions of the Understanding.*

| *Partial.* | *General.* | *Primitively Partial, but tending to become General.* |
|---|---|---|
| Delirious conceptions. | Depression of the intelligence. | Dissociation of ideas. |
| Uncontrollable impulses. | Exaltation. | Abolition of the intelligence. |
| Hallucinations. | | |

*The Elementary Lesions of the Understanding may Exist.*

1. With preservation of the reason.
2. Accompanied by insanity.

*Insanity, the Consequence of the Lesions of the Understanding.*

Two kinds of insanity characterized by

| The first, | The second, |
|---|---|
| By the loss of consciousness of the lesions of the understanding. | By the simple inability of the will to resist certain impulses. |

---

### Special Pathology.

*Forms of Mental Diseases.*

| SIMPLE FORMS: *Curable.* | SIMPLE FORMS: *Incurable.* | MIXED FORMS: |
|---|---|---|
| Monomania (partial lesions). | Incoherent dementia (dissociation of ideas). | Combinations of the curable forms, or of curable forms with incurable. |
| Melancholia (general lesion: depression). | Simple dementia (abolition of ideas). | |
| Mania (general lesion: excitation). | | |
| Insanity of double form (depression and excitation succeeding each other regularly in the same patient). | | |

*Mental Diseases,*

| *Owing to a specific Cause.* | *Associated with Cerebral Affections, following upon or symptomatic of these Affections.* |
|---|---|
| Delirium tremens. | 1. General paralysis. |
| Delirium produced by belladonna, datura, haschich, &c. | 2. Convulsive affections, epilepsy, hysteria, chorea. |
| | 3. Local organic affections of the brain. |

*Appendix.*

Imbecility, Simple with cretinism.

These tables sufficiently explain the leading principles of M. Baillarger's classification, and dispense us from the task of following him through a minute exposition of the various heads. We, however, think it useful to draw attention to the three partial elementary lesions of the understanding, as distinguished by our author, a distinction, we believe, descending from Esquirol. Uncontrollable impulses ought, in strict propriety, to be distinguished from lesions of the understanding. Springing as they do from a lesion of the moral affections, and existing not seldom in conjunction with more or less integrity of the intellectual faculties, it seems (although the objection is psychological, and therefore not one, we suppose, that would weigh with our author) improper to place it between two lesions in which the intellect simply is affected. The distinction between delirious conceptions and hallucinations, conditions usually confounded in this country under the common name of delusions, is important. There is surely something generically different between delirious conceptions—consisting in false ideas, extravagant, ridiculous and absurd, impossible of execution, springing up in the diseased mind of the patient, and not immediately excited by real or supposed impressions upon the senses falsely interpreted—and hallucinations consisting in an apparent perversion or disorder of the organs of sense, in which either sensations are perceived in the absence of all external excitation, or in which certain real excitations of the senses convey to the mind impressions widely different from their real nature. It may be urged that in ultimate analysis both these conditions are resolved into one and the same morbid state, in which the judgment is perverted, and is unable to appreciate correctly the error of ideas and the false suggestions of the senses, and that therefore the common term delusion may properly apply to both. But in practice we still hold that the distinction is useful, and that it deserves to be more attended to than is usual among us.

M. Baillarger is at some pains to defend his description of *depression* and *exaltation* of the intelligence as *general* lesions. We think his view in strict accordance with clinical experience. In those cases marked by depression, and they are the most numerous by far, it is impossible not to observe that the depression tells with a paralysing weight upon all the faculties of the mind. There is no such thing as partial depression. This is a point upon which M. Guislain strongly insists. The celebrated professor of Ghent goes so far as to contend that alienation is truly a grief; that it is primitively a phrenalgia; and that a profound depression is the most constant and universal symptom amongst the insane.

We have nothing to observe relative to the lesions of the understanding, primitively partial but tending to become general. The remaining divisions of the first table, based as they are upon M. Baillarger's fundamental definition, which, being already sufficiently discussed, need not again arrest our attention. We proceed to offer a few reflections upon the second table, which embraces the special pathology of insanity, and which may be said to present the author's opinions, expressed in a clinical and practical point of view. Under the common head of simple forms he preserves the old forms of monomania, melancholia, and mania, without disturbing the notions generally attached to these words. These are all ranged together as curable. His division of dementia into incoherent, marked by dissociation of ideas ; and simple, marked by abolition of ideas (although the first form is often but the prelude or transition state into the second), is useful. His third division of "Mixed Forms," expressly framed to find a place for those common but anomalous cases which refuse to obey the laws of systematic nosologists, exhibiting characters which perplex by their variety and seeming incompatibility, displays, we ought not to say, the variety of classifications, but the necessity of circumscribing our expectations as to the fruit to be gathered from them.

In his division of mental diseases owing to a specific cause—in which M. Baillarger includes delirium tremens, and the delirium caused by certain poisons—we think he might also have placed with propriety the delirium arising from fever, specifying those forms of fever, as typhoid and others, which are evidently associated with toxæmia. The relation of this form of delirium to those connected with more obvious poisoning of the blood is manifest. It cannot with propriety be classed along with those forms of insanity which are associated with local organic affections of the brain. It may be quite true that in many cases of fever, meningitis, or other cerebral lesions, may supervene, but these are not primary and essential characters. In the majority of cases of ordinary fever, especially of those that terminate in recovery, the cerebral affection is not organic, but functional, and the consequence of disordered nutrition or unnatural excitation, arising from the altered state of the blood.

We now arrive at another important distinction much insisted upon by M. Baillarger. He divides mental alienations into *idiopathic* and *symptomatic*. In every case, he says, the determination of the question, whether it be idiopathic or symptomatic, should be aimed at. He even says that, *with very rare exceptions*, the distinction may be made even at the outset of the disease. This proposition would appear rather startling, were we not to hasten to explain the precise meaning M. Baillarger attaches to the words idiopathic and symptomatic. The signification in which he uses the word symptomatic is indeed sharply defined, but it is somewhat arbitrary. We need not tarry in order to extract from our author a definition of idiopathic. He nowhere states it precisely. But he defines symptomatic with great precision: so that by a simple process of elimination we may call everything that is not symptomatic, idiopathic—"Those mental alienations are symptomatic which are so of some other cerebral affection." Were he to stop at this point, we think he would hardly be able to justify his dogma that, even at the outset of any case of mental disease, it is easy to decide whether it be idiopathic or symptomatic. Surely we are not yet so far advanced in our knowledge of cerebral pathology as to be able, even in the great majority of instances, to pronounce with certainty upon the presence or absence of cerebral complications. Still less frequently are we in a condition to decide whether—a particular cerebral lesion being discovered, or supposed to be discovered—that lesion was the causative antecedent of the associated mental disorder. But these difficulties are summarily pushed aside by M. Baillarger. He excludes all affections of the heart, stomach, urinary passages, and of all other organs, except of the brain, as having any pretension to be the foundation of symptomatic mental diseases. We cannot here pursue our objection to this exclusion. But we may state, as the result of daily clinical observation, that many mental disorders in women, some rising to all the significance of actual insanity, are truly symptomatic of primitive disease in the organs of generation. Unless the word be wrested forcibly from its usual acceptation, we cannot see why these affections are not entitled to be called symptomatic. But the fact is, M. Baillarger does affix to the word a meaning of his own, and he tells us very intelligibly what that meaning is. He takes no account of any other "cerebral affections but *those which are revealed by disorder in the muscular system.*" He even specifies the kinds of muscular disorder,—namely, general paralysis, epilepsy, hysteria, and chorea. But even this strict limitation is far from removing all occasions of difficulty and error. With the single exception of general paralysis, it cannot be admitted that the muscular disorders are invariably symptomatic of cerebral disease. The attempt, therefore, to link any given cases of mental alienation to cerebral disease, by means of concomitant lesions of the muscular system, signally fails. It appears to us that it would be better to take the larger and less defined signification of the word symptomatic, and to preserve a second place as an *asylum ignorantiæ* for those numerous cases in which our best-exerted skill is baffled in the attempt to trace any relation between the mental alienation and physical disorder. We prefer, on this ground, the division of M. Guislain into idiopathic, symptomatic, and sympathetic. We have thought it the more necessary to state these difficulties that stand in the way of M. Baillarger's classification, because the able physician of the Salpêtrière informs us that his registers at that asylum are constructed upon the principle he has laid down. He keeps two registers. In the one are inscribed all the insane who are affected with different lesions of the locomotive system, as general paralysis, epilepsy, chorea, hysteria; the other chronicles "all those who present none of the above lesions, and who may *for that reason be considered at once as affected with simple or idiopathic alienation.*" When at any future time account is taken of, or reference made to, M. Baillarger's statistics—and M. Baillarger is a laborious statistician—this fact must be borne in mind. We have now given a full exposition of M. Baillarger's classification of mental diseases. If we have been unable to adopt it unreservedly, if we have felt ourselves compelled to urge the difficulties in the way of its acceptation that occur to us, we are at the same time anxious to express our undiminished regard for the great talents, the vast experience, and admirable candour of the author. He has failed in a task which has stimulated many noble ambitious minds, but in which success would, *primâ facie*, appear to be impossible. He has made that clearer which was clear before—that psychology, both physiological and pathological, is not yet sufficiently advanced to admit of the full application of the inductive method. The classification of mental diseases will long continue to baffle the strongest intellects and the most accomplished physicians.

---

